# The importance of phase drift correction for accurate MR thermometry in long duration MR-HIFU exposures

**DOI:** 10.1186/2050-5736-3-S1-P57

**Published:** 2015-06-30

**Authors:** Chenchen Bing, Charles Mougenot, Robert Staruch, Elizabeth Ramsay, Alain Schmitt, Juha Kortelainen, Julius Koskela, Rajiv Chopra

**Affiliations:** 1 University of Texas Southwestern Medical Center, Dallas, Texas, United States; 2 Philips Healthcare Canada, Toronto, Canada; 3 Philips Research, Dallas, Texas, United States; 4 Sunnybrook Health Sciences Centre, Toronto, Canada; 5 Sunnybrook Research Institute, Toronto, Canada; 6 VTT Technical Research Centre of Finland, Tampere, Finland; 7 Philips Healthcare, Vantaa, Finland

## Background/introduction

In MRI-guided high-intensity focused ultrasound (MR-HIFU) therapy, temperature-dependent proton resonance frequency (PRF) shift is a key factor to quantify and visualize the spatial heating pattern in treated and surrounding tissue. However, during treatment, multiple scanner-related changes can impact the accuracy of the temperature measurements obtained with the PRF shift method and cause an over/under estimation of temperature, which can be a major safety issue for treatments involving real-time temperature control. Hence, it is necessary to apply corrections to ensure accurate temperature measurements during heating. Prior to image acquisition, the central MR frequency F0 can be measured to adjust the F0 of next image acquisition. After acquisition, corrections can be applied to the acquired images to remove scanner-related influences, most importantly phase drift. Different phase drift correction algorithms such as conventional and polynomial adaptive drift correction estimate the background phase by fitting a linear or polynomial to the image phase outside the treatment area and perform the correction accordingly. The goal of this study was to understand the performance of these algorithms for long heating durations as would be experienced during hyperthermia or transurethral HIFU (>20 minutes).

## Methods

Data was collected in phantom, animal and human studies ongoing within our research program using both Philips Achieva 3.0T and Ingenia 3.0T MR scanner (Philips Healthcare, Netherlands). Data-sets included heating and no heating, as well as non-invasive and minimally-invasive HIFU devices. MR thermometry with echo-planar imaging (EPI) and conventional gradient echo (FFE) pulse sequences were investigated, with varying repetition time (TR) and EPI factor. Several drift correction algorithms were evaluated, including conventional correction, zero and higher order polynomial adaptive correction, dynamic F0 stabilization by the scanner, and the default drift correction of the clinical MR-HIFU system. Raw data with no correction was analyzed as well. Data acquisition ranged from 5 to 30 minutes, representing the type of acquisition that would be used during hyperthermia or transurethral HIFU.

## Results and conclusions

The average temperature change measured due to drift was approximately 2.6°C per min without drift correction for an EPI sequence while close to 0°C per min for an FFE sequence. The temperature change decreased to 0.1°C per min under conventional drift correction and even further using adaptive drift correction and dynamic F0 stabilization. With longer TR (50ms *vs*. 33ms) and larger EPI factor (15 *vs*. 11), the temperature change decreased to 1.7°C per min and 1°C per min separately. *In vivo* data (rabbit) indicated an average change of 3°C per min (range from 3°C to 3.5°C per min) and was significantly reduced under all four algorithms. During the clinical prostate HIFU treatments a drive of 3°C per min was observed. Image-shifts in the phase encode direction of approximately 1 pixel (1mm) every 10 minutes were measured in the absence of dynamic stabilization. In conclusion, drift corrections is necessary for accurate thermometry during long duration HIFU exposures.

**Figure 1:**
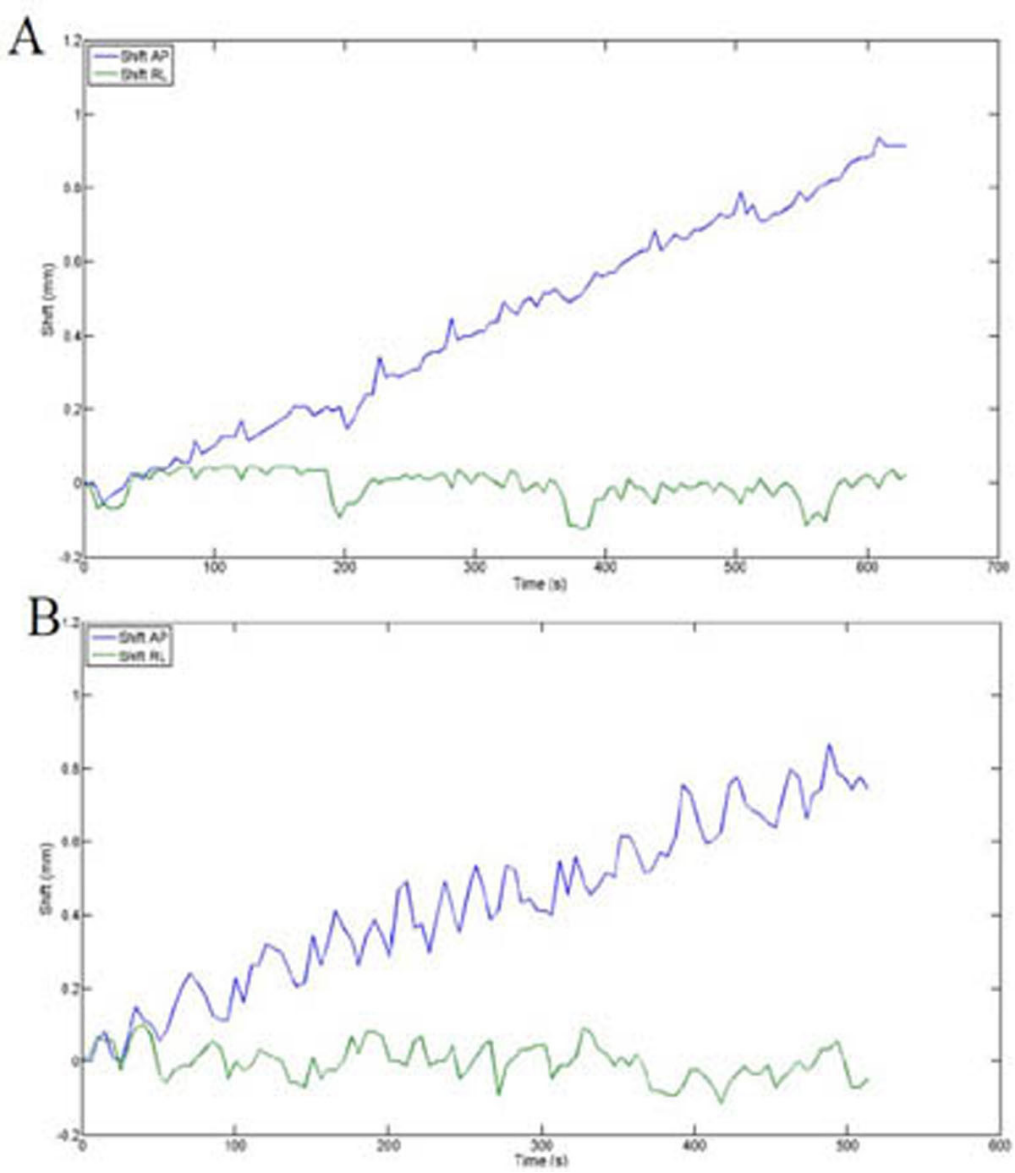
Image shift during transurethral prostate HIFU treatment. Panel A and B represents two patients separately. The blue curve indicates displacement of 0.1mm/min along AP direction (phase encoding direction). The green curve shows no significant displacement along RL direction.

